# Filaria specific antibody response profiling in plasma from anti-retroviral naïve *Loa loa* microfilaraemic HIV-1 infected people

**DOI:** 10.1186/s12879-018-3072-2

**Published:** 2018-04-04

**Authors:** Ghislain Donald Njambe Priso, Abel Lissom, Loveline N. NGU, Nadesh N. Nji, Jules Colince Tchadji, Thibau Flaurant Tchouangueu, Georgia E. Ambada, Carole Stéphanie Sake Ngane, Brigitte Laure Dafeu, Larissa Djukouo, Inès Nyebe, Suzanne Magagoum, Apeh Alfred Ngoh, Ouambo Fotso Herve, Rosario Garcia, Anna Gutiérrez, Arinze S. Okoli, Charles O. Esimone, Flobert Njiokou, Chae Gyu Park, Alain Bopda Waffo, Godwin W. Nchinda

**Affiliations:** 1 Laboratory of vaccinology/biobanking, CIRCB, Messa, Yaounde, Cameroon; 2 Department of animal biology and Phisiology, University of Yaounde I, Yaounde, Cameroon; 3 Department of Microbiology, University of Yaounde I, Yaounde, Cameroon; 4 Department of biochemistry, University of Yaounde I, Yaounde, Cameroon; 5 Department of biochemistry, University of Dschang, Yaounde, Cameroon; 6 Department of biomedical sciences, University of Dschang, Dschang, Cameroon; 7 CSCB (Centre de santé catholique de Bikop), Bikop, Cameroon; 8 Department of medical laboratory sciences, University of Buea, Buea, Cameroon; 9 Department of Biological Sciences, College STEM, 1627 Hall Street, Montgomery, AL 36101 USA; 10 Center for NanoBiotechnology Research, 1627 Harris Way, Montgomery, AL 36104 USA; 11 Laboratory of Immunology, Brain Korea 21 PLUS Project for Medical Science, Severance Biomedical Science Institute, Yonsei University College of Medicine, Seoul, 03722 Republic of Korea; 12 GenØk - Centre for Biosafety, Tromsø, Norway; 13 Department of Pharmaceutical Microbiology & Biotechnology, Nnamdi Azikiwe University, Awka, Nigeria

**Keywords:** HIV-1, *Loa loa*, Microfilaraemia, Loaisis, African eye worm

## Abstract

**Background:**

In West and Central Africa areas of endemic *Loa loa* infections overlap with regions of high prevalence of human immunodeficiency virus type 1 (HIV-1) infections. Because individuals in this region are exposed to filarial parasites from birth, most HIV-1 infected individuals invariably also have a history of filarial parasite infection. Since HIV-1 infection both depletes immune system and maintains it in perpetual inflammation, this can hamper *Loa loa* filarial parasite mediated immune modulation, leading to enhanced loaisis.

**Methods:**

In this study we have assessed in plasma from asymptomatic anti-retroviral (ARV) naïve *Loa loa* microfilaraemic HIV-1 infected people the filarial antibody responses specific to a filariasis composite antigen consisting of Wbgp29-BmR1-BmM14-WbSXP.

The antibody responses specific to the filariasis composite antigen was determined by enzyme linked immunosorbent assay (ELISA) in plasma from ARV naïve *Loa loa* microfilaraemic HIV-1 infected participants. In addition the filarial antigen specific IgG antibody subclass profiles were also determined for both HIV-1 positive and negative people.

**Results:**

Both *Loa loa* microfilaraemic HIV-1 positive and negative individuals showed significantly higher plasma levels of IgG1 (*P* < 0.0001), IgG2 (P < 0.0001) and IgM (P < 0.0001) relative to amicrofilaraemic participants. A significant increase in IgE (P < 0.0001) was observed exclusively in *Loa loa* microfilaraemic HIV-1 infected people. In contrast there was a significant reduction in the level of IgG4 (*p* < 0.0001) and IgG3 (P < 0.0001) in *Loa loa* microfilaraemic HIV-1 infected individuals.

**Conclusions:**

*Loa loa* microfilaraemia in ARV naïve HIV-1 infected people through differential reduction of plasma levels of filarial antigen specific IgG3, IgG4 and a significant increase in plasma levels of filarial antigen specific IgE could diminish *Loa loa* mediated immune-regulation. This in effect can result to increase loaisis mediated immunopathology in antiretroviral naive HIV-1 infected people.

## Background

*Loa Loa,* or the African eye worm, is a human filarial parasite endemic in West and Central Africa, with over 13 million people currently infected with the parasite [1, 2]. During *L. loa* infection (Loiasis) the adult filarial parasite can live in subcutaneous tissues for up 17 years [3] and is capable of producing microfilariae (MF) in peripheral blood which are the further transmitted by the dipteran vector (chrysops spp). The great majority of people infected with *Loa loa* show little or no visible symptoms [4, 5]. This is because *L. loa,* like all helminth infections, modulates immune responses to reach a safe parasite-host equilibrium, where either high levels of *loa loa* microfilaria (microfilaraemic loiasis) or no apparent microfilaria (amicrofilaraemic loiasis) can be found in peripheral blood [6, 7]. Some reports have suggested that elevated levels of filarial parasite antigen specific IgG4 antibody subclass is essential for asymptomatic infection [8]. But other reports have suggested that increased expression of nonspecific polyclonal IgE [9] and elevated levels of specific IgG4 [10] have been associated with loaisis. Nevertheless all antibody isotypes, including IgM, IgE and IgG, are known to play a major role in mediating protection to filarial infections [11].

In sub-Saharan Africa, areas of intense endemic helminth infections overlap with regions of high prevalence of HIV-1 [12]. Therefore the risk of concomitant infection with filarial parasites and HIV-1 is high. In regions of high *L. lao* endemicity like Central and West Africa with concurrent high HIV-1 prevalence, few studies have looked at the interaction between these two infections. Both *L. loa* and HIV-1 are chronic infections where the pathogen can persist for longer periods in the absence of sterilizing immunity. HIV-1 infection depletes and maintains the immune system in a sustained inflammatory state, rendering the host more susceptible to other infectious diseases. In contrast filarial parasites in the majority of cases modulate the host’s immune response to ensure their survival, thereby maintaining asymptomatic infection with elevated plasma levels of parasite antigen specific IgG4 [10]. This is generally associated with a generalized state of hypo-responsiveness which ensures long term filarial parasite survival [11].

In this study, we have investigated the profile of filarial antigen specific antibody responses of *Loa loa* microfilaraemic ARV naïve HIV-1 infected people in comparison with HIV-1 negative participants. This is because filarial parasites employ both filarial antigen specific and nonspecific immunomodulatory strategies to escape elimination from the human host. However ARV naïve HIV-1 infection depletes the immune system and with concomitant persistent *loa loa* microfilaraemia the profile of filarial antigen specific antibody responses is not known. The immune depletion and sustained inflammation associated with HIV-1 infection might alter immune modulation and change the profile of filarial antigen specific antibody responses in the context of microfilaraemic loaisis. Thererfore, the profiles of filarial antigen specific antibody responses in *Loa loa* microfilaraemic HIV-1 infected people could reveal possible interactions between these two infections.

Using a mixture of four recombinant filarial parasite antigens, consisting of Wbgp29, BmR1, BmM14 and WbSXP, we have assessed antibody responses in both microfilaraemic and amicrofilaraemic long standing ARV naïve HIV-1 infected people in the CIRCB AFRODEC cohort [13]. Antibody assays based on Bm14, WbSXP, Wbgp29 or BmR1 have previously been demonstrated to show excellent sensitivity (> 90%) in the detection of filarial specific antibodies [14, 15]. Bm14 and WbSXP, for example, are highly reactive with their respective antibodies in plasma from microfilaraemic individuals. More so prior studies have reported the Bm14 antibody test as a sensitive marker for infection or heavy exposure to filarial parasites [14, 16, 17]. This implies that this mixture of recombinant filarial antigen is suitable for assessing *Loa loa* specific antibody responses.

The consequence of ARV naïve HIV-1 infection was a significant increase in plasma levels of filariasis composite antigen specific IgE. This was in association with a significant decrease in serum levels of filarial antigen specific IgG4 and IgG3, clearly suggesting a reduction in *Loa loa* mediated immunomodulation.

## Methods

### Study population

Participants were 21 to 65 years old and samples were collected as part of the CIRCB AFRODEC study. The CIRCB AFRODEC (African HIV-1 dendritic cell targeted vaccine) cohort study was conducted in Cameroon from November 2012 to November 2016 and enrolled anti-retroviral therapy (ARV) naïve HIV-1 infected people. During the course of 4 years 199 participants out of the 766 members of CIRCB AFRODEC cohort who repeatedly complained of filarial infection were recruited for this study. In addition confirmed 99 HIV-1 negative participants complaining for microfilarae infecions were also monitored alongside their HIV-1 positive counterparts as controls. Finally 25 *Loa loa* microfilaraemic antiretroviral therapy (ARV) naïve HIV-1 infected participants who remained *Loa loa* positive during the course of 4 years were included in the analysis. 25 amicrofilaraemic ARV therapy naïve HIV-1 infected individuals with no signs of loaisis were also included. Similarly a control group of HIV-1 negative individuals was separated into 25 *Loa loa* microfilaraemic and amicrofilaraemic participants. In addition to people who did not provide consent, participants who had been diagnosed with Hepatitis B virus, Hepatitis C virus, Dengue virus, Mycobacterium tuberculosis, or malaria were excluded from the study. Absolute numbers of helper CD4^+^ T cells for HIV-1 positive participants was determined in fresh whole blood by BD multitest CD3/CD8/CD45/CD4 and TruCount tubes (BD Biosciences, San Jose, USA) according to the manufacturer’s instructions. Plasmatic HIV-1 viral load was determined on the m2000rt machine using the Abbott Real-Time HIV-1 Assay protocol.

### Study design (Fig. 1)

Members of the CIRCB AFRODEC cohort who are either HIV-1 positive or negative, were included based on the presence or absence of *Loa loa* microfilaraemia during a period of 4 years. Recruited microfilaraemic participants are those who showed at least two microfilaraemia per year during the period of the study. Amicrofilaraemic participants were Loa loa negative throughout the 4 years of the study.

**Fig. 1:**
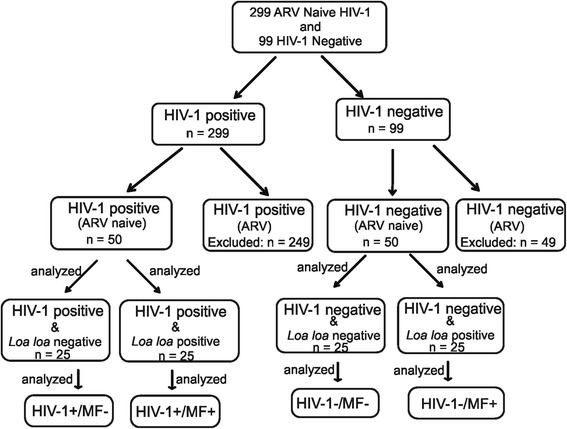
Study flow displaying screening and recruitment of study participants. Members of the CIRCB AFRODEC cohort which are either HIV-1 positive or negative were included based on the presence or absence of *Loa loa* microfilaraemia during a period of 4 years. Recruited microfilaraemic participants are those showed at least two microfilaraemia per year during the period of the study

### Sample collection and processing

Blood was collected into 4-ml plastic vacuum blood spray-coated K2EDTA tubes (Vacutestkirma, Italy). All samples were stored at room temperature and processed within 4 h of collection. To obtain plasma, samples were centrifuged at 2000 rpm for 10 min at 4 °C. The plasma fraction was harvested in sterile conditions under the hood, aliquoted in small, single-use volumes and stored at − 20 °C until use. All plasma obtained from anti-retroviral naive HIV-1 infected participants was heat inactivated for 30 min at 56 °C prior to ELISA assay.

### Filarial diagnosis

Parasitological examination of individuals was done by microscopic examination of 10% Giemsa stained peripheral blood (10 μl), both as thin and thick films. In the CIRCB AFRODEC cohort screening for *Loa loa* microfilaraemia was performed three times annually for each participant. Three independent confirmations of *Loa loa* infection by different researchers were necessary for a sample to be considered positive.

### Enzyme linked immunosorbent assay (ELISA) for IgM, IgG and IgE

Filariasis composite antigen Wbgp29-BmR1-BmM14-WbSXP was diluted in PBS and 100 μl containing 100 ng/well were added to high binding 96-well flat bottom microsorp (Thermo Fisher Scientific) ELISA plates and incubated overnight at 4 °C. The following day, plates were washed three times with PBST (PBS with 0.05% Tween-20) and blocked either with 3% BSA or 1× Roti block (Carl ROTH, Karlsruhe, Germany) for 1 hour at 37 °C. After an additional washing step, 100 μl/well of plasma diluted (1:500) in PBS was added into corresponding wells in triplicates and incubated for 2 hours at 37 °C.

Next the plates are washed five times as described above. Then 100 μl horseradish peroxidase (HRP)-conjugated anti-human IgG (1:2000) and horseradish peroxidase (HRP)-conjugated anti-human IgG1, IgG2, IgG3, IgG4, or IgM (1:4000) antibodies were added, and the plates incubated for 1 h at 37 °C. Similarly for the detection IgE, HRP-conjugated anti-human IgE diluted 1:1000 was used. Antibody isotypes and subclasses used in this study were mouse anti-human IgG Fc (clone JDC-10), mouse anti-human IgM (clone UHB), mouse anti-human IgE Fc (clone B3102E8), mouse anti-human IgG1 Fc (clone HP6001), mouse anti-human IgG2 Fc (clone 31-7-4), mouse anti-human IgG3 Hinge (clone HP6050) and mouse anti-human IgG4 pFc’ (clone HP6023), all HRP conjugate all procured from Southern Biotech® (Birmingham, USA). Plates were then washed five times and 100 μl of 2,2′-Azino-bis (3-ethylbenzthiazoline-6-sulfonic acid) or ABTS one Component HRP substrate was added to each well and incubated in the dark for 30 min. The enzyme substrate reaction was stopped by adding a stop solution (Southern Biotech, Birmingham,USA). The optical densities (OD) at 405 nm were read using a multiscan Fc Elisa microplate reader (Thermo-scientific, USA). All plasma was tested in triplicate and the mean OD values were determined.

### Statistics

Statistical analyses were done using the software GraphPad PRISM version 6.0 (Graphpad Software). Comparisons between groups were by the non-parametric Mann-Whitney-U test (for paired data), Student’s t-test (for unpaired data) and Kruskal-Wallis test. Viral load was expressed on a log10 scale. *P* values of 0.05 or less were considered significant.

## Results

### Study population

199 ARV naïve HIV-1 infected individuals from the CIRCB AFRODEC cohort and 99 HIV-1 negative people who repeatedly complained of filarial infection were monitored for 4 years. During 4 years eligible participants were separated into 4 groups based on their HIV-1 status and persistent Loa loa microfilaraemia. Excluded during the course of the study were individuals diagnosed occasionally with *Loa loa* microfilaraemia, Hepatitis B virus, Hepatitis C virus, Dengue virus, Mycobacterium tuberculosis and malaria. Participants retained for both HIV-1 positive and negative groups based on persistent Loa loa microfilaraemia or amicrofilaraemia were analyzed. Amongst the HIV-1 infected individuals, 25 were HIV-1 positive without microfilaraemia (HIV+/MF-) and 25 were Loa loa microfilaraemic (HIV+/MF+). As shown in Table 1 for the HIV + MF-, 10 (40%) were males and 15 (60%) were females. Regarding HIV + MF+ participants 09 (36%) were males and 16 (64%) were females respectively. In addition 25 microfilaraemia negative (HIV-/MF-) and 25 microfilaraemic (HIV-/MF+) HIV-1 negative participants who were repeatedly tested as part of the CIRCB AFRODEC cohort were analysed as controls. The age distribution between the four different groups was similar (Table 1).

**Table 1:** Study population characteristics

Variable	HIV^−^/MF^−^ (*n* = 25)	HIV^−^/MF^+^ (n = 25)	HIV^+^/MF^−^ (n = 25)		HIV^+^/MF^+^ (n = 25)	
Gender	Male and Female	Male and Female	Male	Female	Male	Female
Participants (%)	8 (32) 17 (68)	11 (44) 14 (56)	10 (40)	15 (60)	9 (36)	16 (64)
Median Age (IQR)	34 (21-41)	49.50 (45.25-55.25)	34 (32-43.50)	37 (27-46)	38 (28-46)	35.5 (28.75-45)
Mf count (MF/ml) Median	N/A	1800 (10-4800)	N/A		986.7 (1-3200)	
Median CD4 counts (cells/mm^3^)	N/A	N/A	499 (IQR 310.5-886.8)		367.5 (IQR 157.8-460.8) ^*^	
Median Viral load (Log_10_ copies/ml)	N/A	N/A	3.05 (IQR 1.76-3.85)		5.04 (IQR 4.530-5.5) ^*^	

*P < 0.05, N/A = Not Applicable, IQR = interquartile range, ns = not significant, MF^+^ = microfilaria positive

As shown in Table 1, microfilaria negative HIV-1 infected participants differed significantly (*p* < 0.05) in their median helper CD4^+^ T cell counts from microfilaraemic HIV-1 infected people (compare 499 with 367.5 cells/mm^3^). In addition plasma viral load in *Loa loa* microfilaraemic HIV-1 infected individuals was significantly higher (*P* < 0.05) than in microfilaraemia negative HIV-1 infected participants.

### *Loa loa* microfilaraemia results in enhanced plasma levels of Filariasis composite antigen specific IgM in both HIV-1 negative and positive individuals

**Fig. 2:**
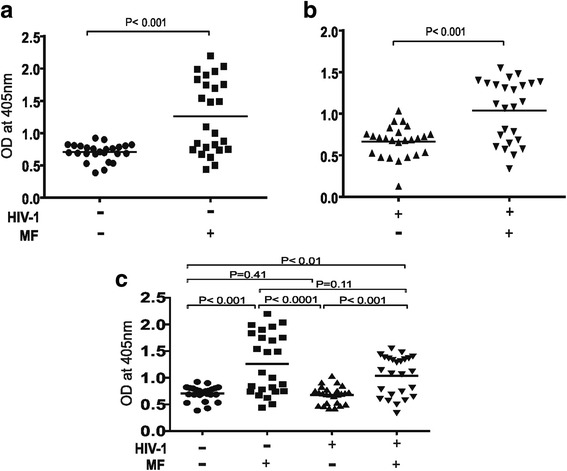
Plasma levels of Filariasis composite antigen specific IgM in *Loa loa* microfilaraemia positive and negative ARV naïve HIV-1 infected people compared to seronegative people. As shown in (**a**) & (**b**) *loa loa* microfilaraemia results in significantly (*P* < 0.001) higher plasma levels of IgM in both HIV-1 negative and positive individuals (compare HIV^−^/MF^+^ with HIV^+^/MF^+^). In (**c**) a comparative statistically analysis is made between the four groups with respect to plasma levels of their Filariasis composite antigen specific IgM. In (**c**) a slight but insignificant reduction (*P* < 0.115) is observed in plasma levels of antigen specific IgM in *Loa loa* microfilaraemic ARV naïve HIV infected people. Statistical analysis was done with prism graph path 6.0, unpaired t test, Mann-Whitney-U tests and Kruskal-Wallis test

When plasma levels of filariasis composite antigen specific IgM were considered both *Loa loa* microfilaraemic HIV-1 negative (in Fig 2a compare HIV^−^/MF^−^ with HIV^−^/MF^+^) and positive (Fig 2b compare HIV^+^/MF^−^ with HIV^+^/MF^+^) individuals showed significantly higher plasma levels (*P* < 0.001) vis a vis microfilaraemia negative individuals (Fig 2 a & b). Loa loa microfilaraemia in both HIV-1 positive and negative individuals was associated with significantly elevated OD values of filariasis composite antigen specific IgM implying a continuous exposure to filarial antigens. The OD values showed a slight negative correlation with the microfilaria burden (*r* = − 0.084 data not shown). In Fig 2c a statistical analysis is shown for the four different groups with respect to plasma levels of filariasis composite antigen specific IgM. When HIV-1 positive and negative individuals were compared, except for a slight reduction in the plasma level of antigen specific IgM of *Loa loa* microfilaraemic ARV naïve HIV-1 infected people there was no significant difference in plasma level of filariasis antigen specific IgM in both microfilaraemia HIV-1 positive and negative individuals (Fig 2c). Thus elevated plasma levels of filarial antigen specific IgM are associated with *Loa loa* microfilaraemia in both HIV-1 negative and positive people.

### ARV naïve HIV-1 infections results in elevated plasma levels of filariasis composite antigen specific IgE exclusively in *Loa loa* microfilaraemic individuals

**Fig. 3:**
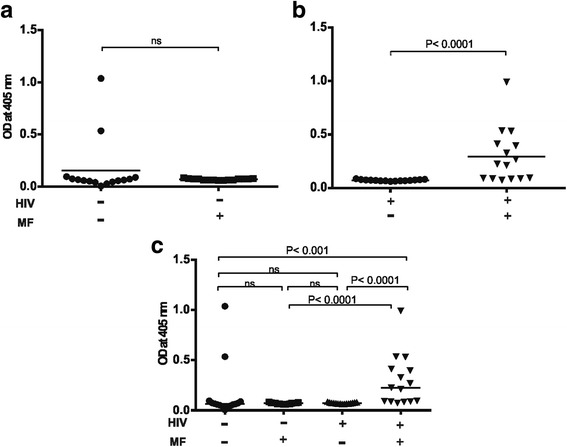
Plasma levels of Filariasis composite antigen specific IgE in *Loa loa* microfilaraemia positive and negative ARV naïve HIV-1 infected people compared to seronegative people. As shown in (**a**) & (**b**) *Loa loa* microfilaraemia results in significantly (P < 0.001) higher plasma levels of antigen specific IgE exclusively in ARV naïve HIV-1 infection (compare HIV^−^/MF^+^ with HIV^+^/MF^+^). In (**c**) a comparative statistically analysis is made between the four groups with respect to plasma levels of their Filariasis composite antigen specific IgE. In (**c**) a significantly higher (*P* < 0.0001) plasma levels of antigen specific IgE is observed exclusively in *Loa loa* microfilaraemic ARV naïve HIV infected people. Statistical analysis was done with prism graph path 6.0, unpaired t test, Mann-Whitney-U test and Kruskal-Wallis test

A unique situation was observed with plasma levels of filariasis composite antigen specific IgE where significantly (*P* < 0.0001) elevated plasma levels of antigen specific IgE was observed exclusively for *Loa loa* microfilaraemic ARV naïve HIV-1 infected people. In contrast little or no antigen specific IgE was detected in the other three groups (compare Fig. 3 a&b). In Fig. 3c a statistical analysis is shown for the four different groups with respect to plasma levels of filariasis antigen specific IgE. Thus ARV naïve HIV-1 infection escalates plasma levels of filariasis antigen specific IgE, which could result in an increase in immunopathology.

### Filariasis composite antigen specific IgG antibody responses in *Loa loa* microfilaraemic antiretroviral naïve HIV-1 infected individuals

**Fig. 4:**
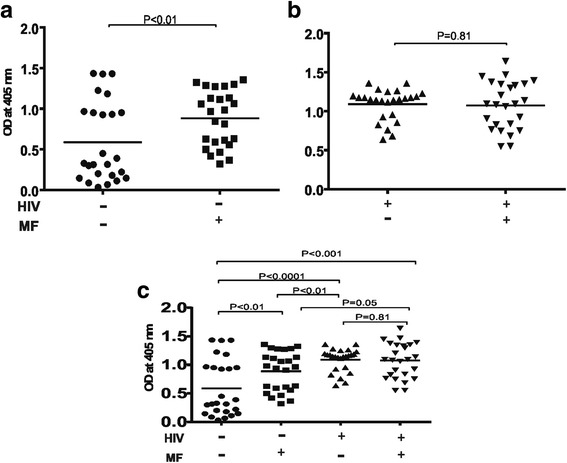
Plasma levels of Filariasis composite antigen specific IgG in *Loa loa* microfilaraemia positive and negative ARV naïve HIV-1 infected people compared to seronegative people. As shown in (**a**) & (**b**) *Loa loa* microfilaraemia results in significantly (P < 0.001) higher plasma levels of antigen specific IgG exclusively in HIV-1 negative individuals (compare HIV-/MF- with HIV-/MF+). In (**c**) a comparative statistically analysis is made between the four groups with respect to plasma levels of their Filariasis composite antigen specific IgG. Statistical analysis was done with prism graph path 6.0, unpaired t test Mann-Whitney-U tests and Kruskal-Wallis test

With respect to plasma levels of filariasis composite antigen specific IgG; *Loa loa* microfilaraemic HIV-1 negative individuals showed significantly higher levels (*P* < 0.01) relative to amicrofilaraemia individuals (in Fig. 4a compare HIV^−^/MF^−^ with HIV^−^/MF^+^). Plasma levels of filariasis composite antigen specific IgG in ARV naïve HIV-1 infected participants in contrast did not differ significantly between *Loa loa* microfilaraemia positive and negative individuals (Fig. 4b compare HIV^+^/MF^−^ with HIV^+^/MF^+^).

In Fig 4c a statistical analysis is shown for the four different groups with respect to plasma levels of filariasis composite antigen specific IgG. During this study ARV naïve HIV-1 infected people generally demonstrated significantly higher plasma levels (*P* < 0.001) of filariasis composite antigen specific IgG relative to HIV negative individuals irrespective of *Loa loa* microfilaraemia. In this case when microfilaraemia negative seronegative individuals are compared with ARV naïve HIV-1 infected people a significantly higher plasma level of filariasis antigen specific IgG was observed for the HIV-1 infected people (Fig. 4c). Increased filariasis specific IgG levels in ARV naïve HIV-1 infected people could be an indication of the absence of effective immunodulation which is relevant in the long term survival of microfilaria. This in effect could escalate filarial parasite mediated immunopathology.

### Increased plasma levels of Filariasis composite antigen specific IgG1 and IgG2 antibody subclass responses in *Loa loa* microfilaraemic individuals

**Fig. 5:**
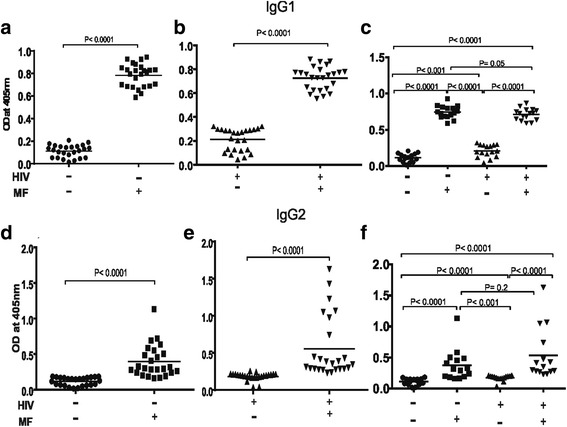
Plasma levels of Filariasis composite antigen specific IgG1 and IgG2 in *Loa loa* microfilaraemia positive and negative ARV naïve HIV-1 infected people compared to seronegative people. As shown in (**a**, **b**&**c**) *Loa loa* microfilaraemia results to significantly (P < 0.001) higher plasma levels of antigen specific IgG1 in both HIV-1 negative and positive individuals (compare HIV-/MF- with HIV-/MF+). Similarly in (D, E &F) *Loa loa* microfilaraemia results to significantly (P < 0.001) higher plasma levels of antigen specific IgG2 in both HIV-1 negative and positive individuals (compare HIV^−^/MF^−^ with HIV^−^/MF^+^). Statistical analysis was done with prism graph path 6.0, unpaired t test, Mann-Whitney-U tests and KruskalKruskal-Wallis test

The plasma level of filariasis composite antigen specific IgG1 was significantly (*P* < 0.0001) elevated during *Loa loa* microfilaraemia in both HIV-1 positive and negative individuals (Fig. 5a&b). In Fig. 5c a statistical analysis is shown for the four different groups with respect to plasma levels of filariasis composite antigen specific IgG1. When participants were compared with respect to HIV-1 status a significant increase in plasma level of IgG1 was observed in microfilaraemia negative ARV naïve HIV-1 infected individuals relative to seronegative individuals. In contrast when microfilaraemic HIV-1 positive and negative individuals were compared a significant reduction (*P* < 0.05) in IgG1 was observed in ARV naïve HIV-1 infected participants (Fig. 5c). This implies that *Loa loa* microfilaraemic ARV naïve HIV-1 infection can lead to a significant reduction in plasma levels of filariasis composite antigen specific IgG1.Similarly *Loa loa* microfilaraemia resulted in elevated plasma levels of IgG2 in both HIV-1 positive and negative individuals (Fig. 5d&e). When HIV-1 infected people were compared with their negative counter parts no significant difference (*P* < 0.2) was observed between microfilaraemic HIV-1 positive and negative people with respect to plasma levels of filariasis composite antigen specific IgG2 (Fig. 5f).

### Reduction in plasma levels of Filariasis composite antigen specific IgG3 and IgG4 antibody subclass responses in *Loa loa* microfilaraemic ARV naïve HIV-1 infected individuals

**Fig. 6:**
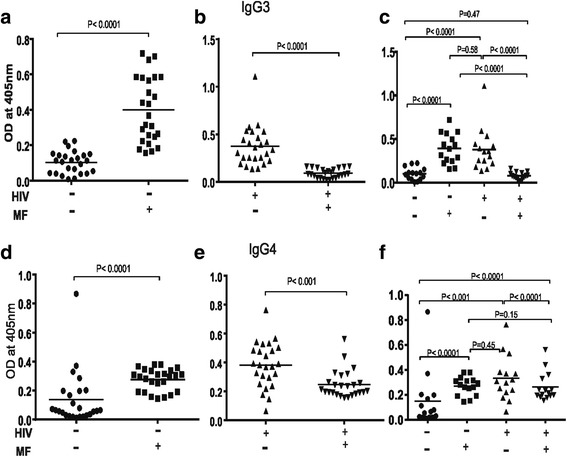
Plasma levels of Filariasis composite antigen specific IgG3 and IgG4 in Loa loa microfilaraemia positive and negative ARV naïve HIV-1 infected people compared to seronegative people. As shown in (**a**) *Loa loa* microfilaraemia results to significantly (P < 0.0001) elevated plasma levels of antigen specific IgG3 in HIV-1 negative people on the one hand and a significant reduction (P < 0.0001) of antigen specific IgG3 in HIV-1 positive individuals on the other hand. Similarly in (**e**) *Loa loa* microfilaraemia results to significantly (P < 0.0001) higher plasma levels of antigen specific IgG4 in HIV-1 negative individuals in contrast to a significant reduction (P < 0.001) in HIV-1 positive individuals (compare HIV-/MF- with HIV-/MF+). Here the effect of *Loa loa* microfilaraemic HIV-1 infection was a significant reduction in plasma levels of filariasis composite antigen specific IgG3 (P < 0.0001) and IgG4 (P < 0.001). Statistical analysis was done with prism graph path 6.0, unpaired t test, Mann-Whitney-U tests and Kruskal-Wallis test

Plasma level of filariasis composite antigen specific IgG3 was significantly (*P* < 0.0001) higher in *Loa loa* microfilaraemic HIV-1 negative individuals (Fig. 6a). In contrast in ARV naïve HIV-1 infected people there was a significant reduction in plasma levels of filariasis composite antigen specific IgG3 (Fig. 6b). In Fig. 6c a statistical analysis is shown for the four different groups with respect to plasma levels of filariasis composite antigen specific IgG3. When participants were next compared with respect to HIV-1 status a significant reduction in plasma level of IgG3 was associated with microfilaraemic ARV naïve HIV-1 infection. This was in contrast to microfilaraemia negative individuals where significantly high plasma levels of filariasis composite antigen specific IgG3 was observed in ARV naïve HIV-1 infected individuals (Fig. 6c). On the other hand filariasis composite antigen specific IgG4 Plasma levels were reduced significantly (P < 0.0001) in *Loa loa* microfilaraemic HIV-1 infected individuals compared to microfilaraemia negative HIV-1 positive people. In addition microfilaraemia negative HIV-1 infected people showed comparatively similar filariasis composite antigen specific IgG4 Plasma levels like *Loa loa* microfilaraemic HIV-1 negative individuals. (Fig. 6 d&e). In Fig. 6f a statistical analysis is shown for the four different groups with respect to plasma levels of antigen specific IgG4. Comparing HIV-1 negative with positive individuals filariasis composite antigen specific IgG4 Plasma levels were generally higher in ARV naïve HIV-1 positive than negative.

## Discussion

In this study we have assessed the impact of ARV naïve HIV-1 infection on the patterns of filariasis composite antigen specific antibody responses in microfilaremic loaisis by analyzing plasma profiles of filariasis composite antigen specific IgG, IgM and IgE amongst HIV-1 positive and negative participants. This is mainly because filairial antigen specific IgG, IgE and IgM have been demonstrated to play critical roles in acquired immunity to filarial parasite infection [18]. However in the context of microfilaraemia in ARV naïve HIV-1 infection where there is not only HIV-1 driven immune depletion but equally sustained inflammation little is known about the profiles of filarial antigen specific antibody responses. The need for this study becomes pertinent especially with recent reports indicating that the hitherto relatively less pathogenic loaisis [4, 5] can be a significant cause of mortality in *Loa loa* endemic countries [19]. Two main groups consisting of ARV naïve HIV-1 infected members of the CIRCB AFRODEC cohort and HIV-1 negative participants were used for this study. The ARV naïve HIV-1 infected group was further split into *Loa loa* microfilaraemia positive (HIV^+^MF^+^) and negative (HIV^+^MF^−^) individuals respectively. Similarly the HIV-1 negative group consisted of microfilaraemia (HIV^−^MF^+^) positive and negative (HIV^−^MF^−^) individuals. All participants were members of the CIRCB AFRODEC cohort for the last 4 years and were repeatedly monitored for *Loa loa* microfilaraemia during this period.

Using a filariasis composite antigen consisting of Wbgp29-BmR1-BmM14-WbSXP in ELISA assay differences in filarial antigen specific antibody responses between the four subgroups was analyzed based on the presence or absence of microfilariae. Our study revealed significantly elevated plasma levels of filariasis composite antigen specific IgG1, IgG2 and IgM in plasma from both microfilaraemic ARV naïve HIV-1 positive and negative participants. These findings agree with previous studies which linked elevated levels of filarial antigen specific IgG1 and IgG2 with the presence of microfilariae [20]. The HIV-1 infection status had a significant effect on the plasma level of filariasis composite antigen specific IgE, IgG3 and IgG4 in Loa loa microfilaraemic individuals. Thus, while in HIV-1 negative people *Loa loa* microfilaraemia resulted to a significant increase in plasma levels of filarial antigen specific IgM, IgG, IgG1, IgG2, IgG3 and IgG4, it produced a completely different outcome in ARV naïve HIV-1 infected people. In this case apart from IgG1 and IgG2 plasma levels of filariasis composite antigen specific IgG3 and IgG4 were significantly decreased in microfilaraemic ARV naïve HIV-1 infection. In addition microfilaraemic ARV naïve HIV-1 infected people also showed an exclusive increased in plasma levels of filarial antigen specific IgE which was significantly higher than all the groups described in this study.

Microfilaraemia during lymphatic filariasis has been linked to elevated levels of species specific IgG4 [21, 22]. Previous studies have shown that *Loa loa* specific IgG4 was elevated in both microfilaraemic and amicrofilaraemic persons [9, 10, 23]. However using the filariasis composite antigen assay we detected significantly higher plasma levels of antigen specific IgG4 in microfilaraemic HIV-1 negative people relative to their HIV-1 positive counterparts. This agrees with previous observation indicating that microfilariae were the main inducers of IgG4 [24]. In this context elevated levels of filarial antigen specific IgG4 have been incriminated in immunomodulation which is necessary for the filarial parasite to evade immune destruction [9, 25–27].

The significant reduction in plasma levels of IgG4 in *Loa loa* microfilaraemic HIV-1 infected people is probably due to immune depletion resulting from the ARV naïve HIV-1 infection. This implies that the reduction in plasma levels of filarial antigen specific IgG4 mediated by ARV naïve HIV-1 infection even in the presence of microfilariae could potentially exacerbate *Loa loa* filarial mediated immunopathology. This is especially so as the decrease in filarial antigen specific IgG4 was accompanied in microfilaraemic HIV-1 positive people by a significant increase in plasma levels of filarial antigen specific IgE. Several reports suggest that the rate of induction, magnitude and the ratio between, the IgE and IgG4 isotypes are major determinants of the clinical outcomes of lymphatic filariasis. In this way, patients with chronic lymphatic disease have been reported with elevated ratios of filarial antigen specific IgE to IgG4. This was in contrast to asymptomatic microfilaraemic patients who presented high plasma levels of IgG4 with a corresponding low IgE to IgG4 ratio. [22, 28–31]. This implies that in microflaraemic ARV naïve HIV-1 infected people the significantly high plasma levels of filarial antigen specific IgE accompanied with reduced specific IgG4 could be an indication of increased immunopathology. Compared to microfilaria negative HIV-1 positive individuals there was enhanced immune depletion in *Loa loa* microfilaraemic ARV naïve HIV-1 infected people. Here Loa loa microfilaraemic ARV naïve HIV-1 infection also resulted in a significant increase in plasma viral load. A two log increased in viral load relative to microfilaria negative participant is a clear indication that dual chronic *Loa loa* microfilaraemia and HIV-1 infection can escalate the expansion of the virus. Thus in depleting the immune system ARV naïve HIV-1 infection can differentially influence anti-filarial antigen specific IgE, Ig3 and IgG4 in microfilaraemic loaisis. On the other hand *Loa loa* microfilaraemia can escalate immune depletion in HIV-1 infection and significantly influence HIV-1 viral load. This bidirectional effect of *Loa loa* microfilaraemia and ARV naïve HIV-1 infection on each other can escalate morbidity and mortality.

## Conclusions

Thus *Loa loa* microfilaraemia in ARV naïve HIV-1 infected people, through differential reduction of plasma levels of filarial antigen specific IgG3, IgG4 and a significant increase in plasma levels of filarial antigen specific IgE, could diminish Loa loa mediated immune-regulation. This can result in an increase in loaisis mediated immunopathology in antiretroviral naive HIV-1 infected people.

## Abbreviations

ARV: Antiretroviral
BSA: Albumin Serum Bovin
ELISA: Enzyme-Linked Immunosorbent Assay
HIV-1: Human Immunodeficiency virus type 1
HRP: Horseradish Peroxidase
Ig: Immunoglobulin
L. loa: Loa loa
MF: Microfilariae
OD: Optical Densities
PBS: Phosphate Buffered Saline
